# Post-traumatic stress disorder in the Ethiopian population dwelling in war-affected communities: a systematic review and meta-analysis

**DOI:** 10.3389/fpsyt.2024.1399013

**Published:** 2024-05-09

**Authors:** Techilo Tinsae, Shegaye Shumet, Gebresilassie Tadesse, Girmaw Medfu Takelle, Gidey Rtbey, Mamaru Melkam, Fantahun Andualem, Girum Nakie, Tesfaye Segon, Selam Koye, Setegn Fentahun, Wondale Getinet Alemu

**Affiliations:** ^1^ Department of Psychiatry, College of Medicine and Health Science, University of Gondar, Gondar, Ethiopia; ^2^ Department of Psychiatry, School of Medicine, College of Medicine and Health Sciences, University of Gondar, Gondar, Ethiopia; ^3^ Department of Psychiatry, Mattu University, Mettu, Ethiopia

**Keywords:** PTSD, internal conflict, trauma, conflict, war

## Abstract

**Background:**

Post-traumatic stress disorder (PTSD) is a significant mental health concern globally, particularly prevalent in populations exposed to war and conflict. This systematic review and meta-analysis aim to examine the prevalence and factors associated with PTSD among the Ethiopian population residing in war-affected communities.

**Methods:**

The review was reported according to the PRISMA guidelines. Related eligible published articles were searched in electronic online databases such as PubMed, Scopus, Web of Science, MEDLINE/PubMed, Scopus, Embase, Science Direct, Web of Science, Google Scholar, and Google, which reported the prevalence and risk factors of PTSD among people dwelling in the war-affected area until January 2024. The relevant data was extracted using a Microsoft Excel spreadsheet. The meta-analysis was conducted using STATA version 11. The estimated pooled prevalence and risk factors were estimated using a random effect model. The potential risk of publication bias was checked using a funnel plot and Egger’s statistical test.

**Results:**

A total of nine published studies with 6107 participants were analyzed in this meta-analysis. The estimated pooled prevalence of PTSD among people living in war-affected areas was 48.4%, with a 95% CI (37.1, 59.8). This study found a higher prevalence of PTSD among women than men. Being female (OR= 2.2, 95% CI: 1.2, 4.3), witnessing a murder of a loved one (OR= 3.0, 95% CI: 1.2, 7.5), depression symptoms (OR= 2.8, 95% CI: 1.4, 5.6), and anxiety symptoms (OR= 3.4, 95% CI: 1.4, 8.0), a close family member killed or seriously injured (OR= 3.1, 95% CI: 1.2, 7.7), a moderate and high perceived threat to life (OR= 3.4, 95% CI: 1.3, 9.1), and poor social support (OR= 4.4, 95% CI: 1.1, 18.7) were associated with post-traumatic stress disorder.

**Conclusion:**

The result of this study shows the high prevalence rate of PTSD in people living in war-affected areas. disparities in PTSD prevalence, with women being at higher risk, and identified risk factors were witnessing the murder of a loved one, experiencing depression and anxiety, and perceived threat to life. Addressing PTSD in war-affected communities requires comprehensive interventions that consider both individual and contextual factors.

**Systematic review registration:**

www.crd.york.ac.uk/PROSPERO/, identifier CRD42024501384.

## Introduction

Post-traumatic stress disorder (PTSD) is a common, disabling disorder that occurs after exposure to traumatic events (1). Post-traumatic stress can cause a pattern of symptoms that includes a delayed response to an acutely stressful and life-threatening situation or event, such as combat exposure during wartime (2). These symptoms could occur either during or immediately after the occurrence of the traumatic event, or even several days later. The symptoms of traumatic events include initially intense fear or instability, horror, nightmares, and hopelessness. Later, the individual could develop a response to traumatic events that are characterized by persistently re-experiencing the traumatic events, avoidance and hyper-arousal, agitation, anxiety, sleep disturbance, and avoidance of the reminder (3–5). These symptoms of PTSD should persist for more than a month and could cause significant functional impairment.

For the last five years, Ethiopia has experienced multiple internal conflicts, deaths, and injuries. Ethiopia’s history of conflict, including the Derg regime, the Eritrean War of Independence, and recent conflicts in regions like Tigray and Amhara has left a legacy of trauma and psychological distress among its population. The cumulative effects of these conflicts have deeply impacted individuals, families, and communities, necessitating comprehensive efforts to address the mental health needs of war-affected populations and promote healing and reconciliation within Ethiopian society (6). According to studies, war-related victims had a higher rate of PTSD (7, 8) than the general population. According to World Health Organization reports, 21.7% of people in conflict situations have PTSD (9). According to a meta-analysis carried out in war conflict-affected areas, the population that has settled in the affected area has a pooled prevalence of PTSD of 23.5% (10). Another systematic review and meta-analysis were conducted in sub-Saharan African countries in conflict-exposed regions, where the pooled prevalence of PTSD was 30% (11), and in a recent cross-sectional survey conducted in Ukraine, the prevalence of post-traumatic stress disorder (PTSD) was found to be 23.5% (12).

The majority of people affected by conflict may experience several traumas such as the death of a loved one or family member, severe physical harm, sexual or emotional trauma, socioeconomic disadvantages, poverty, altered family dynamics, loss of social support, difficulty obtaining education, encountering hostility and racism, and living in an extremely congested area (13–15). PTSD is linked to the risk of developing physical health problems such as sleep disturbances, parenting difficulties, and interpersonal problems and is most strongly associated with comorbid depression, substance use, and suicidal behavior (16, 17). It is also linked with decreased well-being and unemployment when it persists unremittingly for years (18–20).

Various studies were conducted to show the rate and potential risk factors of PTSD in Ethiopia, which range from 19.4% (21) to 67.5% (22), but they demonstrated great variation across geographical settings. Based on this fact, there was a need for nationally representative data on the rate and determinant factors of PTSD among war-exposed individuals in the country. In addition, the existing epidemiological data on post-traumatic stress disorder in residents of conflict-affected areas is desperately needed to assess the precise burden of prevalence and risk factors for PTSD to allocate resources for health care appropriately and effectively. Given the lack of concrete coverage regarding the prevalence and determinants of post-traumatic stress disorder (PTSD) in conflict-affected regions of Ethiopia, this systematic review and meta-analysis aimed to address this gap. The primary objective was to provide an estimation of the pooled prevalence of PTSD and identify determinant risk factors among individuals residing in war-affected communities in Ethiopia.

## Method

### Searching strategy

The detailed protocol of this systematic review and meta-analysis was registered in PROSPERO with a reference number of CRD42024501384. This study was reported according to the preferred Reporting Items for Systematic Review and Meta-analysis (PRISMA) guideline. Before reporting the meta-analysis, the relevant published articles were searched in public electronic databases such as MEDLINE/PubMed, Scopus, Embase, Science Direct, Web of Science, Google Scholar, and Google, which reported the prevalence and risk factors of PTSD among people dwelling in the war-affected area until January 2024. The search process was performed in the English language using appropriate keywords, including post-trauma stress disorder, trauma, post-trauma, stress, conflict, war-affected area, trauma disorder, post-trauma disorder, stress-related disorder, trauma-related disorder, post-conflict, prevalence of PTSD, and determinants of PTSD. In addition, the AND/OR operator was also used to obtain more comprehensive access to all articles. PubMed searching strategies: ((((((((post-trauma stress disorder [title/abstract]) OR post-trauma [title/abstract]) AND stress-related disorder OR trauma-related disorder [title/abstract]) OR war-affected area [title/abstract]) OR PTSD (title/abstract]) OR post-conflict [title/abstract]) AND prevalence of PTSD [title/abstract]) OR determinant of PTSD [title/abstract]) OR Ethiopia.

### Eligibility criteria

#### Inclusion criteria

The studies’ inclusion criteria include observational studies (cross-sectional and survey studies), in which the original published paper reports the prevalence and risk factors of PTD. The population lives in war-affected areas. Eligible literature published in the English language. Literature conducted in Ethiopia, available up to January 2024, met the inclusion criteria.

#### Exclusion criteria

Case reports, interventional studies, qualitative studies, studies without full-text accessibility, editor’s letters, studies with inadequate data, studies unrelated to the topic, review studies, and duplicate studies are among the exclusion criteria in this research.

### Data extraction and quality assessment

Two authors (author one and author four) were individually involved in the extraction of relevant studies from the databases. All the eligible studies were screened. After the selection of eligible studies, study characteristics, and relevant data, the name of the author, publication year, study location and setting, study design, sampling method, sample size, response rate, screening tool, and prevalence were extracted. We contacted authors through email for additional information whenever required. In cases of disagreement between two reviewers, arbitration was made by other authors. The authors used the Newcastle-Ottawa Scale (NOS) (23) critical appraisal checklist adopted for a cross-sectional study. The scale is used to score the articles under three categories: (i) selection (score 0−5); (ii) comparability (score 0−2); and (iii) outcome (score 0−3); the total score range is 0−10. The selection category consists of parameters, such as representativeness of the sample, adequacy of the sample size, non-response rate, and use of a validated measurement tool to gather data on exposure. The comparability category examines whether subjects in different outcome groups are comparable based on the study design and analysis and whether confounding factors were controlled for or not. The outcome category includes whether data on outcome (s) were collected by independent blind assessment, through records, or by self-reporting. The outcome category also includes whether the statistical tests used to analyze the data were clearly described and whether these tests were appropriate or not. Stars were assigned to evaluate study quality: 9–10 stars indicate ‘ very good’ quality, 7–8 stars ‘good’ quality, 5–6 stars ‘satisfactory’ quality, and 0–4 stars ‘unsatisfactory’ quality (23).

### Outcome variables

The primary outcome of this systematic review and meta-analysis was to assess the prevalence and risk factors of PTSD in people dwelling in war-affected areas.

### Heterogeneity and publication bias

The existence of heterogeneity was checked by inverse variance (I^2^) was used to quantify it. The values of statistics of 25%, 50%, and 75% were used to declare low, moderate, and high heterogeneity, respectively (24). The publication bias was checked visually by funnel plot, and objectively using Egger’s statistical test. In addition, a p-value less than 0.05 was used to declare the presence of heterogeneity across studies and publication bias (25). to declare the absence of publication bias. Trim and fill analyses were conducted to manage the publication bias.

### Statistical analysis

The data were retrieved in Microsoft Excel spreadsheet format. The analysis was conducted using STATA version 11 statistical software. The logarithm and standard error of the odds ratio (OR) for each included study were generated using the generate command on STATA. The pooled prevalence of PTSD and its risk factors were presented in the form of a forest plot. The presence of heterogeneity among the included studies was checked by the inverse variance index (I^2^), and the authors considered I^2^ values > 50% to represent significant heterogeneity (26). An estimated pooled prevalence and associated risk factors of PTSD Random effect model were computed to estimate the pooled prevalence and associated risk factors of PTSD among people dwelling in war-affected communities using a forest plot diagram with a corresponding 95% CI and OR. A funnel plot was used to check the presence of publication bias. In addition, Egger’s statistical test was used to check the statistical significance of publication bias. The sensitivity analysis was done with the random effect model to observe the effect of a single study on the overall pooled estimate.

## Results

### Literature searching

**Figure 1:**
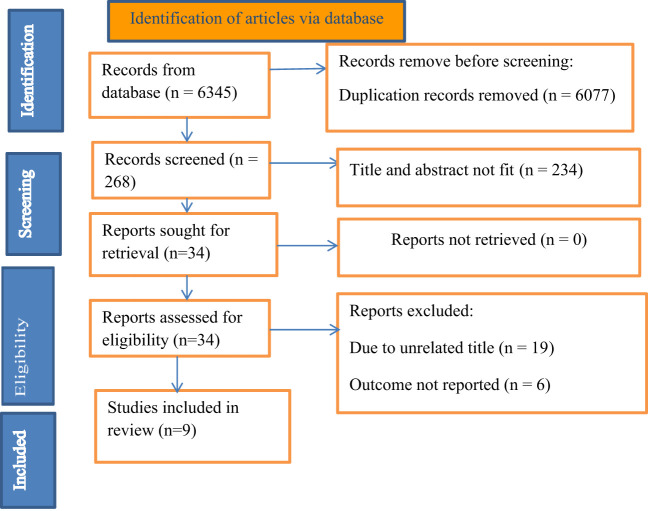
Study selection process.

The search strategy retrieved 6345 articles. After the removal of duplication articles, 268 articles remained. Based on the eligibility criteria 34 full-text articles were accessed for eligibility, 19 were excluded due to unrelated titles, and 6 articles due to lack of outcome interest. Finally, 9 eligible full-text articles were used to analyze this meta-analysis (**Figure 1**).

### Characteristics of the included studies

In this systematic review and meta-analysis, nine primary studies were involved. Among the included studies, eight studies were from the Amhara region (21, 22, 27–31), and one study was from the Southern Nations, Nationalities, and Peoples’ Region (SNNPR) (32) (**Table 1**). All of the articles were published in the most recent four-year period (2020-2023). All of the articles were conducted using a cross-sectional study design. Most of the studies were assessed using the PTSD checklist for DSM-5 (PCL-5) measurement tool. Regarding study settings of the primary study, seven eligible studies were conducted in community-based settings whereas two studies were conducted in internally displaced people (IDP) settings.

**Table 1:** Characteristics of included studies among people dwelling in war-affected areas, in Ethiopia (n = 9).

Authors name	Years	Region	Design	Tool	Mean age	Study population	Setting	Sample size	Prevalence (%)	Prevalence in men (%)	Prevalence in women (%)	Non-response rate (%)
Ali et al., 2022 (28)	2022	Amhara	Cross-sectional	PCL-5	35	war-survivors	Community	610	59.8	46.6	71	98.80
Anbesaw et al., 2022 (21)	2022	Amhara	Cross-sectional	PCL-5	36.01	war-survivors	Community	785	19.4	N/A	N/A	95.60
Bethelehem, 2022	2022	Amhara	Cross-sectional	PCL-5	34	war-survivors	Community	830	63	22.53	40.48	96
Birhan et al., 2023 (31)	2023	Amhara	Cross-sectional	PCL-C	39.87 years	war-survivors	Community	600	34.5	18.5	16	97.60
Biset et al., 2023	2023	Amhara	Cross-sectional	CPSS-SR	8.45	war-survivors	Community	846	36.45	N/R	N/R	94.33
Kassaye et al., 2022	2022	Amhara	Cross-sectional	PCL-5	33.2	war-survivors	Community	597	56.28	53.6	46.4	98
Madoro et al., 2020 (32)	2020	South Ethiopia	Cross-sectional	PCL-5	32.98	war-survivors	IDP	625	58.4	39.2	60.8	98.30
Makango et al., 2023 (22)	2023	Amhara	Cross-sectional	PCL-5	39.12	war-survivors	IDP	406	67.5	47.4	52.6	99
Teshome et al., 2023 (27)	2023	Amhara	Cross-sectional	PCL-5	40	war-survivors	Community	808	40.8	32.1	50.8	99

N/R*= Not reported.

### Qualities of studies

The Newcastle-Ottawa Scale (NOS) was used to assess the quality of the included studies methodologically. In the evaluation, we assessed 9 articles to satisfy the quality assessment in terms of selection, outcome measurement, and statistical test. The risk of bias in each study was assessed using kappa values, which range from 0.88 to 1 (**Table 2**).

**Table 2:** The quality and agreed level of bias and level of agreement on the method qualities include articles in this systematic review and meta-analysis based on a sample, outcome, objective, response rate, and analysis method (n = 9) .

Author, year of Publication	Q1	Q2	Q3	Q4	Q5	Q6	Q7	Q8	Q9	Total score	Kappa value	Level of agreement
Ali et al., 2022 (28)	Y	Y	Y	Y	Y	Y	Y	Y	Y	9	1	Almost perfect
Anbesaw et al., 2022 (21)	Y	Y	Y	Y	Y	Y	Y	Y	Y	9	1	Almost perfect
Bethelehem, 2022	Y	Y	Y	Y	Y	Y	N/R	Y	Y	8	0.88	Almost perfect
Birhan et al., 2023 (31)	Y	Y	Y	Y	Y	Y	Y	Y	Y	9	1	Almost perfect
Biset et al., 2023	Y	Y	Y	Y	Y	Y	Y	Y	Y	9	1	Almost perfect
Kassaye et al., 2022	Y	Y	Y	Y	Y	Y	Y	Y	Y	9	1	Almost perfect
Madoro et al., 2020 (32)	Y	Y	Y	Y	Y	Y	Y	Y	Y	9	1	Almost perfect
Makango et al., 2023 (22)	Y	Y	Y	Y	Y	Y	Y	Y	Y	9	1	Almost perfect
Teshome et al., 2023 (27)	Y	Y	Y	Y	Y	Y	Y	Y	Y	9	1	Almost perfect

Y, yes; NR, not reported.

Question codes:

1. Was the sample frame appropriate to address the target population?

2. Were study participants sampled in an appropriate way?

3. Was the sample size adequate?

4. Were the study subjects and the setting described in detail?

5. Was the data analysis conducted with sufficient coverage of the identified sample?

6. Were valid methods used for the identification of the condition?

7. Was the condition measured in a standard, reliable way for all participants?

8. Was there appropriate statistical analysis?

9. was the response rate adequate, and if not, was the low response rate managed appropriately?

### The estimated pooled prevalence of post-traumatic stress disorder

**Figure 2:**
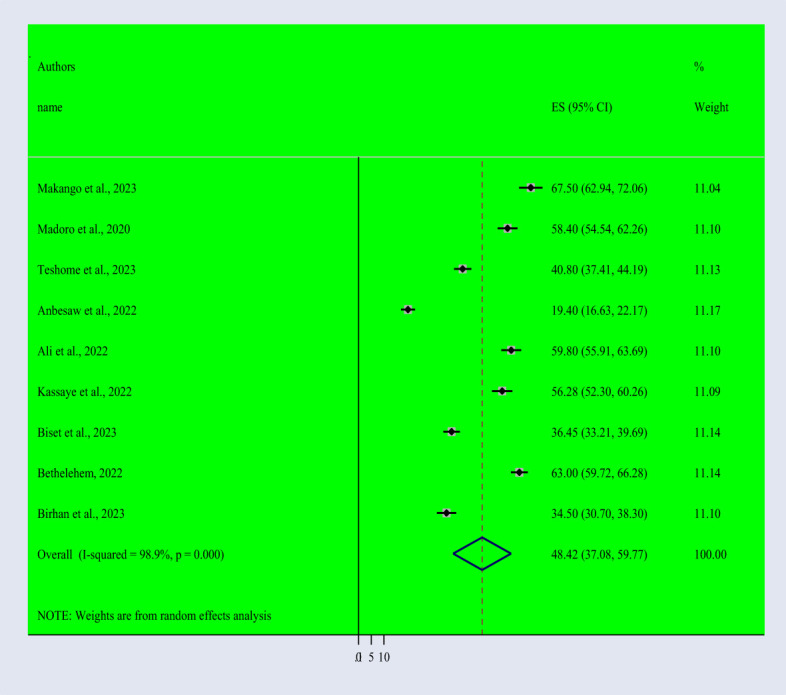
Forest Plot describing the pooled prevalence of PTSD among people dwelling in war-affected areas in Ethiopia with a 95% CI.

**Figure 3:**
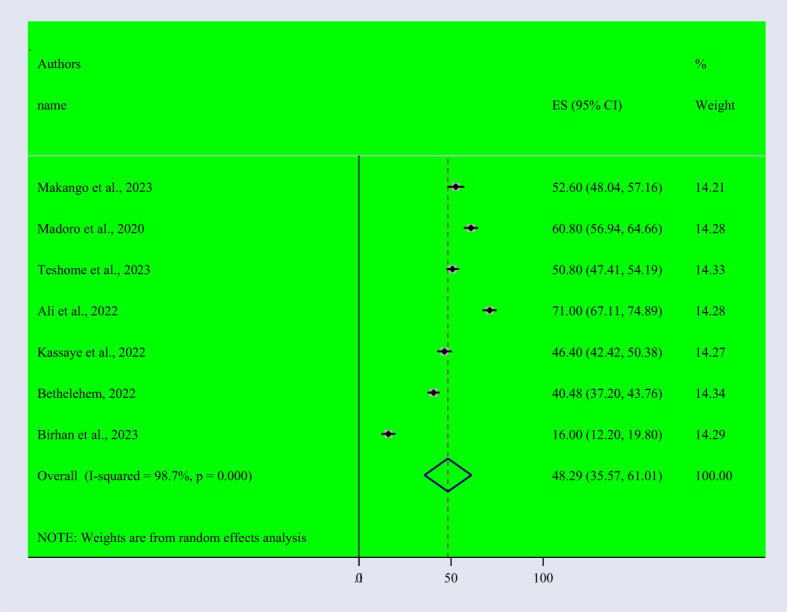
Forest Plot describing the pooled prevalence of PTSD among women dwelling in war-affected areas in Ethiopia with a 95% CI.

**Figure 4:**
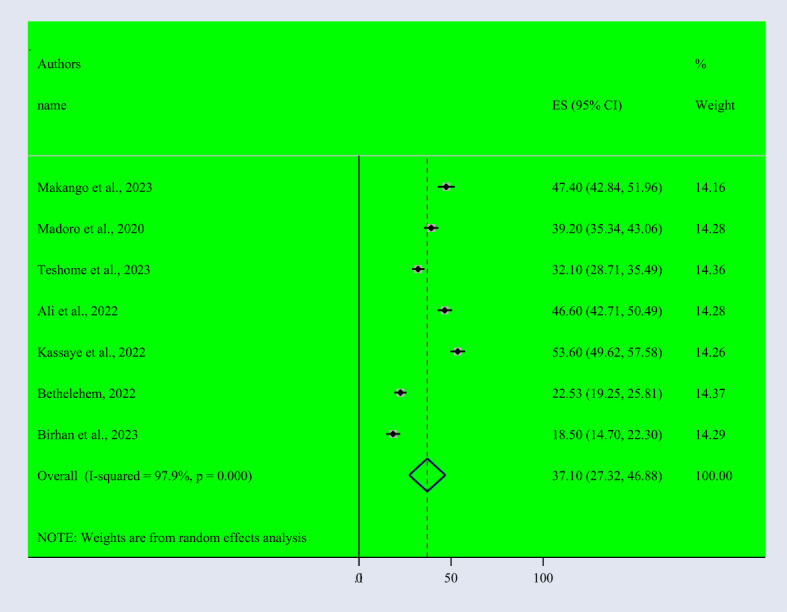
Forest Plot describing the pooled prevalence of PTSD among men dwelling in war-affected areas in Ethiopia with a 95% CI.

Based on the random effect model, the overall pooled prevalence of post-traumatic stress disorder among people dwelling in war-affected areas was 48.42% with a 95% CI (37.08, 59.77) (**Figure 2**). From records of this meta-analysis, we observed the highest prevalence rate of PTSD in women (48.3% with a 95% CI of 35.6, 61.0) (**Figure 3**) than in men (37.1% with a 95% CI of 27.3, 46.9) (**Figure 4**).

### Subgroup analysis

**Figure 5:**
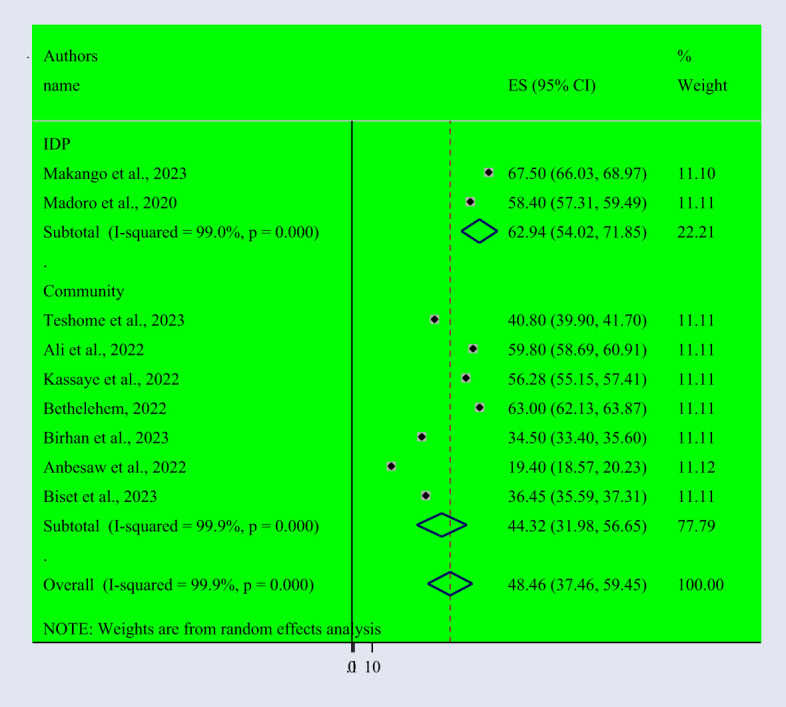
Subgroup analysis based on the study settings of PTSD among people dwelling in war-affected areas.

Subgroup analysis was conducted based on the study setting of the primary studies, which were done in community-based and IDP settings. According to the findings, we observed a higher prevalence of PTSD in IDP settings (62.94% with a 95% CI of 54.02-71.85) than in community-based studies (44.32% with a 95% CI of 31.98-56.65) (**Figure 5**).

### Publication bias and sensitivity analysis

**Figure 6:**
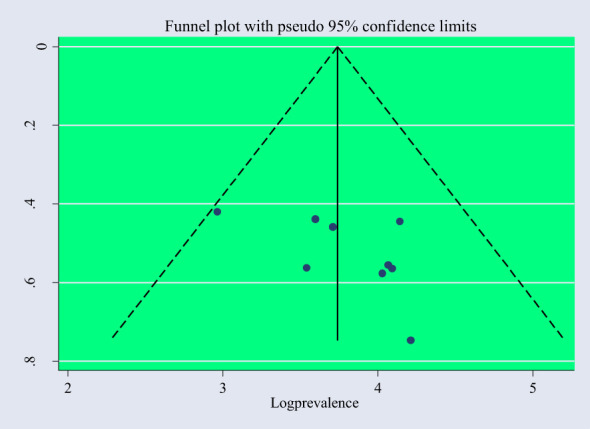
Funnel plot with a pseudo95% confidence interval that describes the heterogeneity of the pooled prevalence of PTSD among people dwelling in war-affected areas in Ethiopia.

**Figure 7:**
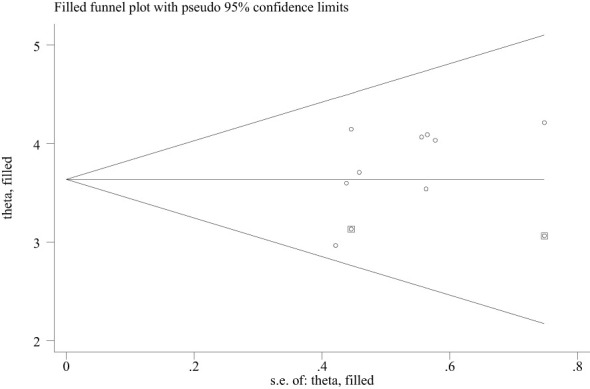
Trim and fill analysis of PTSD among people dwelling in war-affected areas in Ethiopia, 2024.

**Figure 8:**
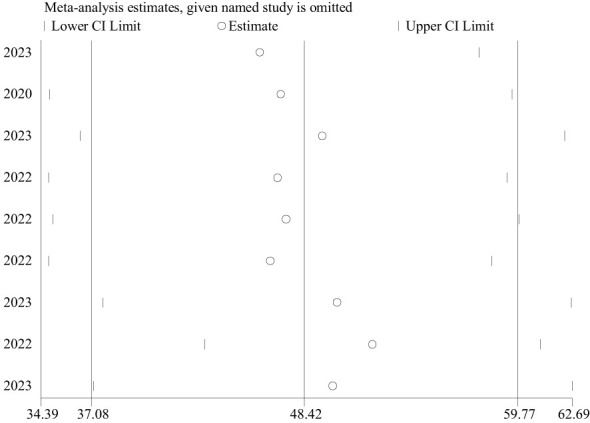
The sensitivity analysis for the pooled estimate of PTSD among people dwelling in war-affected areas in Ethiopia.

The included studies were checked to assess the potential publication bias visually by funnel plot. The funnel plot is a symmetrical distribution. This indicates the absence of publication bias since the included studies fell within the triangular region (**Figure 6**). In addition, the results of Egger’s test showed the presence of publication bias (P-value< 0.05) (**Table 3**). So, we used trim and fill analysis to manage this publication bias (**Figure 7**). The sensitivity analysis was done with the random effect model to observe the effect of a single study on the overall pooled estimate. The result showed that the included did not show significant differences (**Figure 8**).

**Table 3:** Egger’s test of PTSD among people dwelling in war-affected areas in Ethiopia.

Egger’s test					
std_Eff	Coef.	std. Err.	t	P>|t|	[95% conf. Interval]
slope	-41.05735	29.32469	-1.40	0.204	-110.3992 28.28452
bias	48.21622	16.24538	2.97	0.021	9.801992 86.63045

### Factors associated with post-traumatic stress disorder

**Figure 9:**
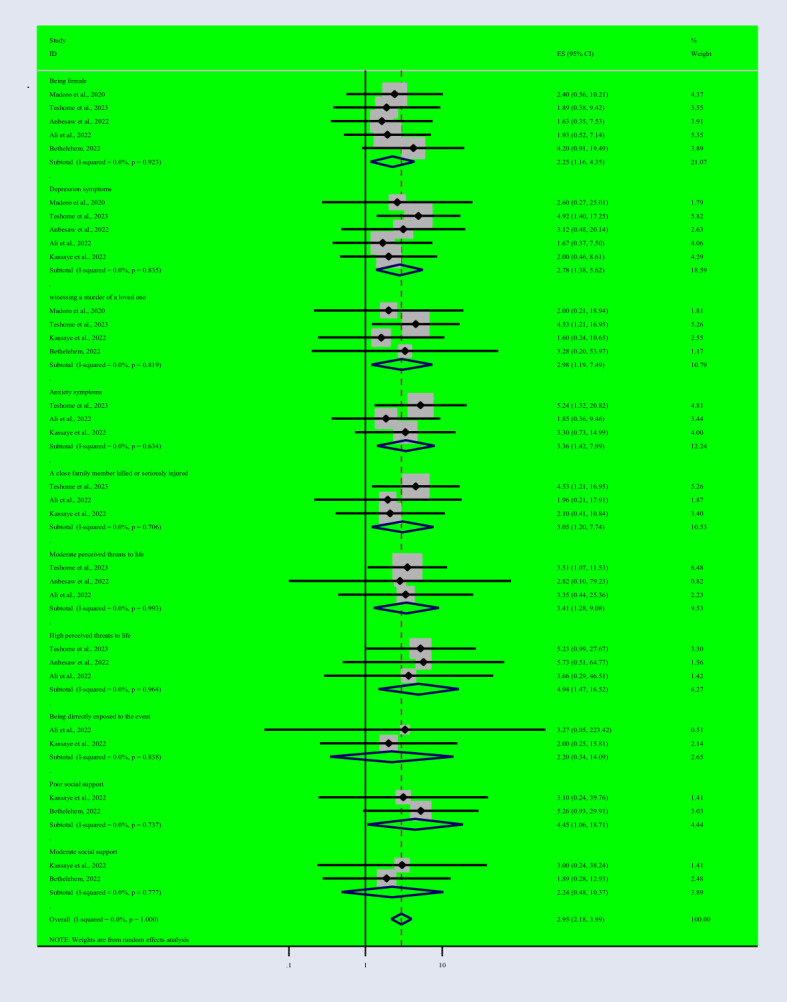
Factors associated with PTSD among people dwelling in war-affected areas in Ethiopia.

Of nine included studies seven studies that conveyed the factors associated with post-traumatic stress disorder among people that dwell in war-affected areas were incorporated in this systematic review and meta-analysis. Based on the random effect model, the following factors were associated with the occurrence of post-traumatic stress disorder among conflict victim individuals. The odds of being female (POR = 2.25: 95% CI: 1.16, 4.35), the odds of having witnessed a murder of a loved one (POR = 2.98; 95% CI: 1.19, 7.49), Conflict victim individuals who had depression symptoms (POR =2.78: 95% CI: 1.38, 5.61) and anxiety symptoms (POR = 3.36; 95% CI: 1.42, 7.99), the odds of a close family member killed or seriously injured (POR = 3.05; 95% CI: 1.21, 7.74), conflict victim individuals who had a moderate and high perceived threat to life (POR = 3.41; 95% CI: 1.28, 9.08) and (POR = 4.94; 95% CI: 1.47, 16.52), conflict victim individual who had poor social support (POR = 4.45; 95%: 1.06, 18.71) were associated with post-traumatic stress disorder (**Figure 9**).

## Discussion

This systematic review and meta-analysis tried to ascertain the concrete pooled evidence on the prevalence and associated risk factors of PTSD among people living in war-affected areas in Ethiopia. For the last six years, Ethiopia has experienced multiple internal conflicts. Even though different scholars attempted to show the prevalence and associated risk factors of PTSD, aggregated pooled evidence was not presented. In our study, around nine relevant, eligible primary studies with 6107 participants were incorporated to conduct this analysis. This meta-analysis showed that the estimated pooled prevalence of PTSD among people dwelling in war-affected areas was 48.42% with a 95% CI (37.08, 59.77). Additionally, our research verified that eight common factors exacerbate the occurrence of PTSD in people dwelling in war-affected areas —being female, having witnessed a loved one’s murder, experiencing symptoms of depression or anxiety, having a close family member killed or gravely injured, feeling that one’s life is in danger, and having inadequate social support—are linked to post-traumatic stress disorder (PTSD). These show that in creating plans to stop and treat PTSD in war-affected communities, related variables need to be taken into account.

According to this meta-analysis, we found a higher prevalence rate of PTSD among women than men living in war-affected areas. The results of this finding are supported by previous meta-analyses done in war-affected areas (33–37). The possible justification for this discrepancy might be the fact that men and women may utilize different coping strategies in response to trauma. Women may be more likely to ruminate on traumatic events or seek social support, while men may be more inclined to use avoidance or substance abuse as coping mechanisms. Societal norms and gender roles can influence how men and women cope with traumatic events. Women may face greater stigma or barriers to seeking help, leading to higher rates of untreated PTSD, or women may experience different types of trauma compared to men during conflicts. They might be more likely to experience sexual violence, which can lead to a higher risk of developing PTSD or hormonal differences between men and women may play a role in the development and manifestation of PTSD symptoms. For example, fluctuations in estrogen levels can affect the stress response and emotional regulation. Therefore, addressing these disparities requires a multifaceted approach that considers gender-specific needs and challenges in mental health support and intervention programs.

The overall pooled prevalence of post-traumatic stress disorder (PTSD) in our study was 48.42%. The finding result of this study is similar to previous meta-analysis studies done on African migrants, accounting for 37.9% (38), Ethiopia at 39% (14), Africa at 55.64% (39), and the meta-analysis conducted among immigration at 42% (40). It suggested that the similarity in prevalence rates across different studies validates the reliability and robustness of the findings, strengthening the evidence base for the prevalence of PTSD in these contexts. This consistency also highlights the urgency of addressing the mental health needs of vulnerable populations, particularly those exposed to trauma in war-affected areas. However, the prevalence of PTSD in the current systematic review is higher than in previous systematic reviews conducted in the war-affected area, with a point prevalence of around 26.51% (41); systematic reviews and meta-analyses carried out among Palestinian children and adolescents exposed to political violence (36% (42);, another systematic review and meta-analysis were carried out in youth exposed to the Syrian crisis; the pooled prevalence of PTSD was 36%, 36.9% (43, 44); and another systematic review was carried out in individuals involved in armed conflict; the pooled prevalence was approximately 31% (45). The possible justification for this discrepancy might be due to the nature of traumatic experiences can vary widely across different conflict-affected populations. For example, populations experiencing systematic persecution, genocide, or mass displacement may be exposed to more severe and pervasive trauma compared to those facing localized conflict or political unrest. The severity and complexity of traumatic experiences can contribute to higher rates of PTSD. The severity of PTSD can be influenced by the intensity and frequency of trauma exposure. Populations experiencing more severe and frequent traumatic events, such as direct combat exposure, prolonged displacement, or mass atrocities, may have higher rates of PTSD compared to those exposed to less severe trauma. The cumulative impact of multiple traumatic events over time can exacerbate the risk of developing PTSD. Populations exposed to ongoing or recurrent violence, displacement, and human rights abuses may experience cumulative trauma, leading to higher rates of PTSD compared to those exposed to isolated traumatic events. Sociocultural norms and beliefs surrounding trauma, mental health, and help-seeking behaviors can vary widely across different populations. The stigma surrounding mental illness, particularly PTSD, may vary, affecting the likelihood of individuals reporting symptoms and seeking treatment. Therefore, considering the severity of traumatic events is essential for understanding the variation in PTSD prevalence estimates across different conflict-affected populations. It highlights the importance of addressing the underlying determinants of trauma exposure and providing comprehensive support and intervention strategies to mitigate the impact of severe trauma on mental health outcomes.

According to the subgroup analysis report a higher prevalence of PTSD was found among participants in IDP settings than in community-based study settings. This finding of this result is supported by previous studies conducted in Africa (46, 47). The possible justification for this discrepancy might be due to trauma exposure which is an individual in IDP settings may have experienced higher levels of trauma due to forced displacement, conflict, or natural disasters compared to those in stable community settings. The ongoing stressors and uncertainties in IDP settings can contribute to a higher prevalence of PTSD. On the other hand, it might be due to social support factors because community-based studies may include individuals with stronger social support networks, which can act as protective factors against developing PTSD. Conversely, IDP settings may lack such support networks due to disrupted social structures and loss of community ties, thereby increasing vulnerability to PTSD. In addition, this might be due to environmental effects hence living conditions in IDP settings, such as overcrowding, lack of necessities, and exposure to ongoing violence or threats, can exacerbate stress levels and contribute to the development or persistence of PTSD symptoms.

Factors associated with this systematic review and meta-analysis: being female was the risk factor for developing post-traumatic stress disorder in people dwelling in war-affected communities. This finding is supported by previous studies (48–54). The possible reason is that women may be more likely to experience certain types of trauma during conflicts than men, such as sexual violence, intimate partner violence, or gender-based discrimination. These types of traumatic experiences can increase the risk of developing PTSD. Sociocultural factors may play a major role in the development of PTSD, such as sociocultural norms and expectations surrounding gender roles can influence how men and women perceive and respond to traumatic events. Women may face additional stressors related to caregiving responsibilities, social roles, and cultural expectations, which can impact their risk of developing PTSD. Biological factors that might be exacerbating the occurrence of PTSD, such as hormonal differences between men and women, can influence the stress response and susceptibility to PTSD. For example, fluctuations in estrogen levels in women may affect the regulation of stress hormones and contribute to increased vulnerability to PTSD.

Among participants, having a history of witnessing the murder of a loved one or being seriously injured increases three times the risk of developing post-traumatic stress disorder in people living in war-affected communities compared to their counterparts. This finding was supported by studies conducted previously (27, 55). The possible reason could be the fact that the severity of the traumatic events may increase the occurrence of PTSD because individuals who have witnessed the murder of a loved one or experienced serious injury often face more severe and direct exposure to traumatic events compared to their counterparts. The intensity and severity of traumatic exposure can significantly impact the risk of developing PTSD, with more severe traumas associated with a higher risk. Also, this might be due to the nature of traumatic events, such as witnessing the murder of a loved one or experiencing serious injury represents traumatic events that involve a high degree of violence, threat to life, and loss. These types of traumas are known to be particularly distressing and are associated with a higher risk of developing PTSD compared to other types of traumatic experiences, or they might be due to survivor guilt; hence, individuals who survive traumatic events in which loved ones are killed or seriously injured may experience survivor guilt, feeling guilty for having survived while others did not, or the reason might be due to psychological effects. Traumatic events involving the murder of a loved one or serious injury can have profound psychological effects, including feelings of helplessness, horror, grief, guilt, and betrayal. These psychological reactions can significantly increase the risk of developing PTSD.

Depression and anxiety were other risk factors associated with PTSD compared to those who did not have depression and anxiety symptoms in war-affected areas. These results are supported by the previous study (56–58). The possible reason might be that individuals with a predisposition to depression and anxiety may also be more susceptible to developing PTSD following exposure to traumatic events. These conditions may interact and exacerbate each other, leading to greater symptom severity and impairment, or they might be due to depression, which may arise from feelings of hopelessness, worthlessness, and loss of interest or pleasure in activities. Anxiety may manifest as persistent worry, hyperarousal, and avoidance of trauma-related stimuli. These psychological responses can co-occur with PTSD symptoms and contribute to the complexity of trauma-related mental health issues, or they might be due to impairment of coping functioning, hence; depression and anxiety symptoms can impair individuals’ ability to cope with traumatic stressors and regulate their emotions effectively. Persistent feelings of sadness, worry, and fear may interfere with adaptive coping strategies and exacerbate PTSD symptoms. Conversely, PTSD symptoms such as intrusive memories, hypervigilance, and avoidance behaviors can exacerbate depression and anxiety.

In addition, individuals who had a moderate and high perceived threat to life were associated with risk factors for PTSD compared with their counterparts in war-affected areas. This might be due to the intensity of traumatic events because a perceived threat to life represents a significant aspect of traumatic experiences in war-affected areas. Individuals who perceive themselves to be in imminent danger or at risk of death during conflict-related events are more likely to experience intense fear, helplessness, and horror, which are core features of PTSD, or it might be due to a sense of control and safety; hence, perceived threats to life can undermine individuals’ sense of control and safety, leading to feelings of vulnerability and powerlessness. The loss of control and perceived inability to protect oneself or loved ones during traumatic events can exacerbate feelings of trauma and increase the risk of developing PTSD. Also, this might be due to the psychological impact of traumatic events because a perceived threat to life can have profound psychological effects, including heightened arousal, hypervigilance, and intrusive thoughts or memories related to the traumatic event. Individuals who perceive a moderate to high threat to their lives may experience persistent anxiety, fear, and distress, which increase the risk of developing PTSD symptoms.

Poor social support had four times higher odds of having post-traumatic stress disorder than those who had strong social support in war-affected areas. This finding is supported by previous studies (59, 60). The possible reason is that social support serves as a buffer against the adverse effects of trauma and stress. Strong social support networks provide individuals with emotional, instrumental, and informational support, which can help them, cope with traumatic experiences and mitigate the risk of developing PTSD. Conversely, poor social support can leave individuals feeling isolated, unsupported, and unable to effectively cope with trauma, increasing their vulnerability to PTSD. Furthermore, this might be due to social support fosters a sense of belonging and connectedness, which are essential for psychological well-being. Individuals with strong social support networks feel valued, cared for, and connected to others, reducing feelings of loneliness and isolation. In contrast, poor social support can exacerbate feelings of alienation, detachment, and disconnection, which are risk factors for the development of PTSD. Overall, the association between poor social support and higher odds of PTSD in war-affected areas underscores the importance of strengthening social support networks and implementing interventions aimed at enhancing social connectedness and support in these communities. Addressing social isolation and promoting social cohesion are essential components of trauma-informed care and mental health support services in war-affected areas.

### Limitation

The findings may not be generalizable to all war-affected populations, as the study focused specifically on Ethiopia. Other countries may have different sociocultural contexts and levels of trauma. Another limitation of this study is that the primary studies included in the meta-analysis may have used different methodologies, assessment tools, and criteria for diagnosing PTSD, leading to heterogeneity in the results. Furthermore, there may be a bias toward publishing studies with significant findings, which could influence the overall prevalence estimate. In addition, the limitation of this study is its narrow scope, which included studies involving only internally conflict-affected individuals. Moreover, all of the studies in this systematic review and meta-analysis had cross-sectional study designs.

## Conclusion

The result of this study shows the high prevalence rate (48.42%) of PTSD in people living in war-affected areas. The research underscores gender disparities in PTSD prevalence, with women being at higher risk. This suggests the importance of gender-sensitive approaches in mental health interventions. Identified risk factors such as witnessing the murder of a loved one, experiencing depression and anxiety, perceived threat to life, and poor social support highlight the complex interplay of individual, social, and environmental factors in PTSD development. Addressing PTSD in war-affected communities requires comprehensive interventions that consider both individual and contextual factors. Strategies should include trauma-informed care, psychosocial support, and efforts to strengthen social support networks. Despite the findings, further research is needed to better understand the nuanced experiences of PTSD in diverse war-affected populations and to develop targeted interventions tailored to their specific needs.

## Data availability statement

The raw data supporting the conclusions of this article will be made available by the authors, without undue reservation.

## Author contributions

TT: Formal analysis, Investigation, Software, Supervision, Validation, Visualization, Writing – review & editing. SS: Investigation, Methodology, Supervision, Visualization, Writing – review & editing. GT: Investigation, Methodology, Validation, Visualization, Writing – review & editing. GM: Methodology, Software, Validation, Visualization, Writing – review & editing. GR: Formal analysis, Investigation, Validation, Writing – review & editing. MM: Methodology, Visualization, Writing – review & editing. FA: Formal analysis, Software, Writing – review & editing. GN: Investigation, Methodology, Visualization, Writing – review & editing. TS: Investigation, Methodology, Writing – review & editing. SK: Formal analysis, Methodology, Writing – review & editing. SF: Investigation, Supervision, Visualization, Writing – review & editing. WG: Formal analysis, Investigation, Methodology, Supervision, Validation, Visualization, Writing – review & editing.
